# Effect of drinking, smoking, and smartphone overdependence on drug addiction among Korean adolescents

**DOI:** 10.3389/fpubh.2025.1524375

**Published:** 2025-04-22

**Authors:** Jaehee Jeong, Wanhyung Lee, Seunghyun Lee

**Affiliations:** ^1^College of Nursing, Seoul National University, Seoul, Republic of Korea; ^2^Department of Preventive Medicine, College of Medicine, Chung-Ang University, Seoul, Republic of Korea; ^3^Department of Convergence Medicine, School of Medicine, Pusan National University, Yangsan-si, Republic of Korea

**Keywords:** alcohol consumption, smoking, smartphone overdependence, drug addiction, interaction, adolescent

## Abstract

**Introduction:**

Adolescent brain development increases vulnerability to drug addiction due to diminished impulse control and the presence of mental health disorders. Alcohol consumption, smoking, and smartphone overdependence have been individually associated with higher drug use, but their combined impact on adolescent drug addiction remains underexplored. This study examines the interaction effects of alcohol consumption, smoking, and smartphone overdependence on drug addiction risk among adolescents.

**Methods:**

Data were obtained from the 16th Korea Youth Risk Behavior Web-based Survey (2020), including 54,948 students from 793 schools. Interaction effects of alcohol consumption, smoking, and smartphone overdependence on drug addiction risk were analyzed, along with a dose-response relationship analysis.

**Results:**

Alcohol consumption, smoking, and smartphone overdependence were significantly associated with increased drug addiction risk. Earlier initiation of drinking and smoking was linked to a higher risk of drug addiction. A significant interaction effect between alcohol consumption and smartphone overdependence on drug addiction was observed.

**Conclusions:**

Alcohol consumption, smoking, and smartphone overdependence significantly elevate the risk of drug addiction among adolescents, with interaction effects exacerbating this vulnerability. Early initiation of drinking and smoking is particularly associated with a heightened addiction risk. A comprehensive understanding of these interaction effects and dose-response relationships is imperative for the formulation of evidence-based, targeted prevention strategies to mitigate adolescent substance use and addiction.

## Introduction

Adolescence is a critical period of intensive brain development, with the prefrontal cortex and serotonin system continuing to mature. However, due to the incomplete development of these regions, adolescents are susceptible to drug addiction, as impulse control is limited (1). This vulnerability is compounded by the higher prevalence of mental health disorder among adolescents with substance abuse issues, including depression and anxiety compared to non-addicted youth (2). Furthermore, research reveals that drug-addicted adolescents are 2.6 times more likely to attempt suicide compared to their non-addicted peers (3). These findings suggest that adolescent drug addiction's severity is even more significant.

Previous studies indicate an association between alcohol consumption and an elevated risk of smoking and drug use (4–6). Additionally, smokers are statistically more inclined to use illicit drugs, including cocaine, heroin, crack, and marijuana (7). Moreover, studies indicate a reciprocal relationship between smoking and drinking, each reinforcing the other and predicting positive attitudes toward drug (8). Several studies implicated that drinking and smoking may act as gateways to drug addiction.

The adoption of smartphones globally has raised concerns regarding smartphone overdependence (9). Smartphone overdependence is defined as a condition in which an individual experiences problematic consequences due to excessive smartphone use, increased salience of the smartphone, and decreased control over its use (10). The phenomenon of smartphone addiction has been shown to replicate the same pattern of substance use disorders, which are characterized by a loss of control due to increased sensitivity of impulse control and reward systems and is thus considered a form of behavioral addiction (11, 12). However, concerns that the stigmatizing effect of the pathological concept of addiction may lead to passive participation in treatment and prevention, and the inclusiveness and neutrality of the term, National Information Agency have led to the adoption of the term overdependence instead of addiction (10). Despite extensive research, there remains a lack of awareness regarding smartphone overdependence during adolescence as a potential factor in exacerbating the risk of drug addiction (13).

Drinking, smoking, and smartphone overdependence share substantial neurobiological mechanisms, involving common brain circuits. Additionally, impulsivity contributes significantly to the early development of these addictive behaviors (14). Moreover, they are consistent in terms of progressing through similar stages, experiencing tolerance and withdrawal, and being characterized by loss of control over the behavior and compulsive maintenance of it (15). Therefore, it is important to investigate the integrated impact of drinking, smoking, and smartphone overdependence on person with drug addiction based on their similar mechanisms.

The hypotheses of this study are that drinking, smoking, and smartphone overdependence impact adolescents with drug addiction. Therefore, we investigate the association between drinking, smoking, and smartphone overdependence and drug addiction among Korean adolescents using data from the Youth Health Behavior Online Survey. Additionally, we evaluate whether these factors have a synergistic effect on the risk of drug addiction.

## Methods

### Data and participants

This study analyzed raw data from the 16th Korea Youth Risk Behavior Web-based Survey (KYRBWS), which was conducted in 2020 to assess the health behaviors of Korean adolescents and was approved by the Korea Disease Control and Prevention Agency. A total of 54,948 students from 793 schools (398 middle schools and 395 high schools) participated through systematic sampling using a multistage stratified cluster sampling design to ensure representativeness across 17 provinces in Korea. Within each selected school, a systematic sampling approach is utilized to randomly select one class per grade level. The students in these designated classes receive standardized instructions from trained teachers, accompanied by official instructional materials, in the computer laboratory. Prior to participation, all eligible students are provided with comprehensive information about the survey protocol. Upon providing voluntary informed consent, they access the KYRBS platform online, authenticate their participation using a unique certification number, and complete the anonymous self-administered questionnaire (16). Research was conducted in accordance with the latest version of the Declaration of Helsinki. After excluding missing data, 54,809 participants were included in this study.

### Main variables

The assessment of lifetime alcohol consumption experience was conducted using the following questions: “Excluding religious ceremonies, rituals, and ancestral rites, have you ever consumed one or more alcohol in your lifetime?” The initial response options were categorized as “yes” or “no”. Lifelong smoking experience was measured using the following questions: “Have you ever smoked even one or two puffs of a cigarette in your lifetime?” Responses were divided into “yes” and “no” on the original response scale (16). This scale comprises 10 items, each rated on a 4-point Likert scale, resulting in total scores ranging from 10 to 40. According to the cutoff points provided by NIA's smartphone overdependence scale, scores equal to or exceeding 23 were regarded as indicative of smartphone overdependence (17). Specifically, scores between 23 and 30 indicate a potential risk group, while scores of 31 or higher represent a high-risk group. The scale has demonstrated good psychometric properties, with high internal consistency (Cronbach's alpha = 0.84–0.92), indicating it is a reliable measure. For the purpose of this study, which is an exploratory examination of the potential risks of smartphone overuse, we defined smartphone overdependence as a score higher than or equal to the potential risk category. The present study defines drug addiction as the habitual substance use that is pathological and leads to addiction. Drug addiction was measured using the following question: “Aside from the purpose of treatment, have you ever habitually used any drugs or substances such as Stimulants, Neuroleptics, Butanone, Binding agents so far?” The responses were divided into “yes” and “no” on the original response scale (16).

### Covariates

Various covariates, such as sex, age, household income, perceived health status, self-rated stress level, sleep duration, and academic grade were included in this study. We selected covariates from previous studies (17, 18) that indicated a link to both drinking, smoking, or smartphone overdependence and person with drug addiction. Age was categorized into two groups (13–15 and 16–18). The responses regarding household income were categorized into five-classes. Perceived health status was assessed by asking individuals to rate their general health status, with response options including “very healthy, healthy, moderate, unhealthy, and very unhealthy”. The self-rated stress level was measured by asking how much stress you usually feel, and the response was divided into “very high, high, moderate, low, very low.” To assess sleep duration, a question on whether sleep was sufficient to relieve fatigue over the past week was used, and responses were classified “very appropriate, appropriate, moderate, inappropriate, very inappropriate.” Academic grade was assessed by inquiring about students' grades in the past 12 months, with response options categorized as “high, upper-middle, middle, lower-middle, low” (16).

### Statistical analysis

The differences in general characteristics according to drinking, smoking, or smartphone overdependence were calculated for each variable using the chi-square test. Odds ratios (OR) and 95% confidence intervals (CI) were calculated using logistic regression model to evaluate the association between person with drug addiction and drinking, smoking, and smartphone overdependence. Dose-response relationship analysis was conducted using age-standardized prevalence ratio (SPR) with a 95% CI. To calculate SPR, age-specific drug addiction prevalence was calculated for each 5-year age group among study participants. The interaction effect between drinking, smoking, or smartphone overdependence on person with drug addiction was demonstrated based on the P-value, and a relative excess risk due to interaction (RERI) and attributable proportion (AP) were represented by 95% CI. All statistical analysis were performed using SAS (version 9.4; SAS Institute, Cary, NC, USA). Two-tailed P-values < 0.05 were considered statistically significant.

## Results

In this study, from a total of 54,416 participants, 393 (1.0%) had experienced drug addiction, comprising 219 (0.8%) men and 174 (0.7%) women. The baseline characteristics according to prevalence of drug addiction are presented in Table 1. The categories with the highest prevalence of drug addiction were: the older age group (16–18) (0.9%), students with “low” household income (2.0%), students with “very unhealthy” perceived health status (7.4%), students with “very high” self-rated stress level (2.4%), and students with “very inappropriate” sleep duration (1.7%). Regarding academic grade, the proportion of person with drug addiction was highest among students with “low” academic achievement (1.1%), followed by those with “high” academic achievement (0.9%).

**Table 1:** General characteristics according to drug addiction.

	**Drug addiction**, ***n*** **(%)**		***p*-value**
	**No**	**Yes**	
Total participants	54,416 (99.0)	393 (1.0)	
**Sex**			0.099
Men	28,050 (99.2)	219 (0.8)	
Women	26,366 (99.3)	174 (0.7)	
**Age (years)**			< 0.0001
13–15	31,348 (99.4)	180 (0.6)	
16–18	23,068 (99.1)	213 (0.9)	
**Household income**			< 0.0001
High	5,945 (99.1)	55 (0.9)	
Upper-middle	15,180 (99.4)	91 (0.6)	
Middle	26,197 (99.4)	160 (0.6)	
Lower-middle	5,858 (99.0)	61 (1.0)	
Low	1,236 (98.0)	26 (2.0)	
**Perceived health status**			< 0.0001
Very healthy	15,027 (99.5)	70 (0.5)	
Healthy	23,153 (99.6)	105 (0.4)	
Moderate	12,192 (99.1)	117 (0.9)	
Unhealthy	3,805 (97.9)	82 (2.1)	
Very unhealthy	239 (92.6)	19 (7.4)	
**Self-rated stress level**			< 0.0001
Very high	4,461 (97.6)	111 (2.4)	
High	13,887 (99.0)	136 (1.0)	
Moderate	24,228 (99.6)	107 (0.4)	
Low	9,838 (99.7)	32 (0.3)	
Very low	2,002 (99.7)	7 (0.3)	
**Sleep duration**			< 0.0001
Very appropriate	5,541 (99.6)	24 (0.4)	
Appropriate	11,171 (99.6)	43 (0.4)	
Moderate	18,496 (99.3)	123 (0.7)	
Inappropriate	13,351 (99.3)	99 (0.7)	
Very inappropriate	5,857 (98.3)	104 (1.7)	
**Academic grade**			0.0002
High	6,636 (99.1)	63 (0.9)	
Upper-middle	13,300 (99.4)	86 (0.6)	
Middle	16,453 (99.4)	102 (0.6)	
Lower-middle	12,585 (99.4)	80 (0.6)	
Low	5,442 (98.9)	62 (1.1)	
**Drinking experience**			< 0.0001
No	36,370 (99.6)	160 (0.4)	
Yes	18,046 (98.7)	233 (1.3)	
**Smoking experience**			< 0.0001
No	48,936 (99.4)	285 (0.6)	
Yes	5,480 (98.1)	108 (1.9)	
**Smartphone**			< 0.0001
**overdependence**			
No	51,339 (99.3)	338 (0.7)	
Yes	3,077 (98.2)	55 (1.8)	

Among the participants who have experienced drug addiction, 233 (1.3%), 108 (1.9%), and 55 (1.8%) have been observed to have drinking experience, smoking experience, and smartphone overdependence, respectively.

Table 2 presents the results of the logistic regression analysis for the risk of person with drug addiction according to drinking, smoking, and smartphone overdependence. After adjusting for sex, age, household income, perceived health status, self-rated stress level, sleep duration, and academic grade, a significantly higher risk of person with drug addiction was identified in the group with drinking experience, smoking experience, and smartphone overdependence [OR 1.946 (95% CI 1.544-2.454), OR 1.946 (95% CI 1.505-2.515), OR 1.578 (95% CI 1.170-2.128), respectively].

**Table 2:** Result of the multiple logistic regression analysis for the risk of drug addiction.

**Variables**		**Odds ratio (95% confidence interval)**
Drinking experience	No	Reference
		Yes	1.946 (1.544–2.454)
Smoking experience	No	Reference
		Yes	1.946 (1.505–2.515)
Smartphone overdependence	No	Reference
		Yes	1.578 (1.170–2.128)

All results were adjusted sex, age, household income, perceived health status, self-rated stress level, sleep duration, academic grade.

**Figure 1:**
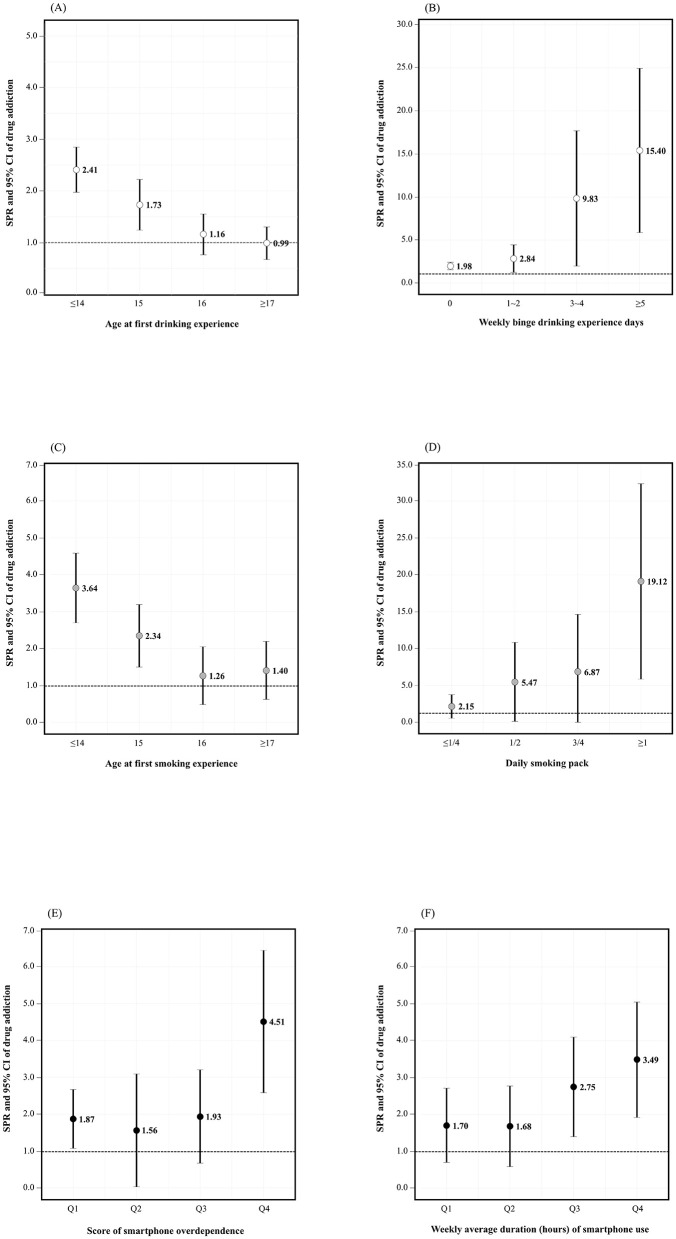
Results of dose-response for drug addiction related with drinking, smoking, and smartphone. SPR and 95% CI for drug addiction **(A)** according to age at first drinking experience, **(B)** weekly binge drinking days, **(C)** age at first smoking experience, **(D)** daily smoking pack, **(E)** score of smartphone overdependence, and **(F)** weekly average duration of smartphone use. SPR, age-standardized prevalence ratio; CI, confidence interval; Q, quartile.

Figure 1 demonstrated age-standardized prevalence ratio (SPR) and 95% CI of person with drug addiction related to drinking, smoking, and smartphone dependence.

The highest SPR for person with drug addiction was observed among individuals who had their first drinking experience before the age of 14, with an SPR of 2.41 (Figure 1A). There was a statistically significant sequential increase in the SPR for drug person with addiction as the number of binge drinking days per week increased. The SPR ranged from 1.98 for those with 0 days to as high as 15.4 for those with more than 5 days of binge drinking per week (Figure 1B). The highest SPR for person with drug addiction was 3.64 for those who had their first smoking experience under the age of 14 (Figure 1C). Furthermore, the SPR increased sequentially with the number of cigarettes smoked per day, with a statistically significant result observed at 1 pack or more (19.12) (Figure 1D). The SPR was statistically highest (4.51) in Q4, when smartphone dependence was highest (Figure 1E). The SPR increased progressively with the average time spent on smartphones per week. The third and fourth quartiles (Q3 and Q4) showed statistically significant increases in SPR, indicating that prolonged smartphone use is associated with a higher risk of person with drug addiction (Figure 1F).

**Figure 2:**
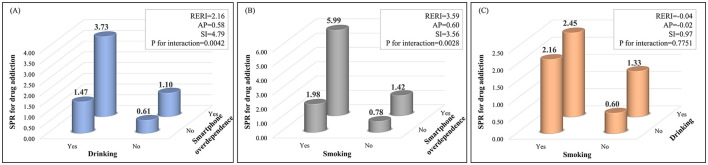
Interaction of drinking, smoking, smartphone overdependence and drug abuse. Interaction effect for drug addiction by SPR between **(A)** drinking and smartphone overdependence, **(B)** smoking and smartphone overdependence, and **(C)** smoking and drinking. SPR, age-standardized prevalence ratio; RERI, relative excess risk due to interaction; AP, attributable proportion; SI, synergic index.

Figure 2 illustrates the results of the interaction analysis between drinking, smoking, and smartphone overdependence on the prevalence of drug addiction. The interaction (SI:4.79) between drinking experience and smartphone overdependence had a significant effect on person with drug addiction (RERI:2.16, AP:0.58, P-value for interaction = 0.0042). The interaction (SI:3.56) between smoking experience and smartphone dependence also had a significant effect on person with drug addiction (RERI:3.59, AP:0.60, *P*-value for interaction = 0.0028). However, the interaction between drinking experience and smoking experience (SI:0.97) had no significant effect on person with drug addiction (P-value for interaction = 0.775).

## Discussion

This study found that drinking, smoking, and smartphone overdependence were more likely to increase person with drug addiction. Notably, interactions between drinking and smartphone overdependence, as well as smoking and smartphone overdependence significantly impacted person with drug addiction. The study revealed the synergistic effects that heightened the risk of person with substance addiction, interactions between alcohol use and smartphone overdependence, and smoking and smartphone overdependence.

The defining feature of addiction is the loss of control and the inability to regulate impulses, despite the individual's awareness of the harmful consequences of their actions. This phenomenon is driven by several neurological factors that interact with one another, including the powerful motivation associated with the activation of the mesolimbic dopamine reward system, the reinforcement of habit formation through changes in neuroplasticity involving learning and memory, and the impaired function of the prefrontal cortex, which is responsible for decision-making and impulse control, influenced by the serotonin and GABA systems (19). Person with behavioral addictions, including person with internet addiction, internet gaming disorder, and gambling, exert effects on the brain's dopamine system, prefrontal cortex function, and amygdala activation that are analogous to those observed in substance addiction (20, 21). The extensive internet capabilities and portability of smartphones afford adolescents broad access to behavioral addictions such as gaming, gambling, and shopping, which may result in internet use disorders (22–24). Engaging in multiple behavioral addictions increases the risk of drug addiction among adolescents with poor self-control and high impulsivity (25–27). Therefore, it is presumed that smartphone overdependence in adolescents who consume alcohol or smoke increases the risk of drug addiction.

The synergistic effects could also be due to the easy access to drugs enabled by smartphones (28, 29). Given the synergistic effects of smartphone overdependence and the increased addiction risks found in this study, it advocates for integrating prevention education on smartphone overdependence with existing efforts aimed at curbing adolescents with drug addiction. Furthermore, emphasizing parental education is crucial, given previous studies highlight the significant influence parents wield over adolescent smartphone dependence and drug addiction (30).

The study revealed that the interaction between drinking and smoking did not significantly influence the prevalence of drug addiction (P for interaction=0.7751). The reason for this may be that the interaction effect was evaluated using drinking and smoking experience as variables. T The study's dose-response findings indicate that drug addiction risk increases with higher levels of smoking, binge drinking, and earlier initiation of smoking and drinking. These results corroborate previous studies linking higher frequencies of drinking and smoking to an increasing possibility of substance use (31).

This study found that age, household income, perceived health status, self-rated stress level, sleep duration, and academic grade influenced adolescent drug addiction. This is consistent with previous studies which have demonstrated that lower household income, poor perceived health status, and high stress levels are associated with an increasing risk of person with drug addiction (32–34). Contrary to previous findings (33), there was no significant difference in the risk of person with drug addiction between genders. This could be attributed to the increased ease of access for purchasing drugs via social media (35). Academic grade was analyzed with a two-tailed distribution, with a prevalence of 0.9% for “high” and 1.1% for “low”. This is consistent with prior research suggesting that higher-achieving students experience extreme academic stress and use substances to cope or to enhance their ability to focus on their studies (33).

Currently, the problem of drug addiction remains a global challenge with considerable social and economic costs (36). Our study highlights a synergistic effect between substance use (alcohol and tobacco) and smartphone overdependence, emphasizing the need to address both substance and behavioral addictions. Furthermore, by demonstrating the multifaceted nature of adolescent addiction risk, our results provide the evidence that can bolster public health initiatives aimed at protecting adolescent development.

A limitation of this study is the potential misinterpretation of the primary outcome, “drug addiction,” due to the wording of the screening question used (“Have you ever habitually used any drugs or substances such as stimulants, neuroleptics, butanone, or binding agents?”). The term “habitual use” broadly indicates regular or repeated use patterns but does not necessarily fulfill clinical diagnostic criteria for drug addiction, which includes dependence, tolerance, withdrawal symptoms, and impaired control. Consequently, some individuals identified as habitual users in this study may not meet the clinical threshold for drug addiction. Additionally, the provided examples (stimulants, neuroleptics, butanone, binding agents) might be interpreted variably, potentially including or excluding substances like cocaine, amphetamines, cannabis, and other volatile solvents. Therefore, caution is advised when interpreting results related to drug addiction, and future studies should employ more clinically precise and validated instruments for assessing substance addiction. The cross-sectional design of the KYRBWS data may limit these findings. Thus, we cannot definitively establish a causal relationship between drinking, smoking, smartphone overdependence and person with drug addiction. A longitudinal study is necessary to reveal any cause-and-effect relationship and to protect adolescents' health. Additionally, while respondents remained anonymous, the online self-administered survey method introduces potential response bias regarding sensitive matters like smoking. Moreover, variables predicated upon subjective assessments, such as household economic status, were included. Furthermore, the utilization of secondary data precluded the inclusion of variables do not present in the original primary dataset.

## Conclusion

This study demonstrates that drinking, smoking, and smartphone overdependence were inclined to increase person with drug addiction. Furthermore, it reveals the interaction between these behaviors and person with drug addiction. These findings provide valuable insight for shaping research and policy, emphasizing the necessity of comprehensive approaches to prevent and intervene in adolescent substance addiction.

## Data Availability

The original contributions presented in the study are included in the article. All data used in this study are publicly available on the KYRBWS website (https://www.kdca.go.kr/yhs/).
